# Serious outcomes of medically attended, laboratory‐confirmed influenza illness among school‐aged children with and without asthma, 2007‐2018

**DOI:** 10.1111/irv.12710

**Published:** 2020-01-14

**Authors:** Huong Q. McLean, Kayla E. Hanson, Allison D. Foster, Scott C. Olson, Sarah K. Kemble, Edward A. Belongia

**Affiliations:** ^1^ Center for Clinical Epidemiology & Population Health Marshfield Clinic Research Institute Marshfield WI USA; ^2^ Minnesota Department of Health St. Paul MN USA; ^3^ Present address: Tennessee Department of Health Nashville TN USA; ^4^ Present address: Hawaii Department of Health Honolulu HI USA

**Keywords:** asthma, child, hospitalization, influenza, pneumonia, vaccination

## Abstract

**Background:**

Asthma was associated with influenza hospitalizations in children during the 2009 pandemic, but it is unclear if asthma is associated with serious illness during seasonal epidemics. Little is known regarding the effect of vaccination on influenza severity in children with asthma.

**Methods:**

Children aged 5‐17 years in a community cohort presenting with acute respiratory illness were prospectively enrolled and tested for influenza from 2007‐08 through 2017‐18 (excluding the 2009‐10 pandemic season). Data from the electronic health record were extracted to determine asthma status and serious outcomes associated with influenza infection. A serious outcome was defined as hospitalization, emergency department visit, and/or pneumonia diagnosis within 30 days of symptom onset. Multivariable logistic regression models were used to assess asthma status and effect of vaccination on odds of a serious outcome.

**Results:**

One thousand seven hundred and sixty four medically‐attended influenza infections among school‐aged children were included. Asthma was confirmed in 287 (16%) children. A serious influenza‐associated outcome occurred in 104 (6%) children. The odds of a serious outcome did not differ between those with confirmed asthma and those without asthma [adjusted odds ratio (aOR): 1.35, 95% confidence interval (CI): (0.77‐2.35), *P* = .3]. The effect of vaccination on serious outcomes was not modified by asthma status [aOR for children without asthma: 0.55 (95% CI: 0.28‐1.07), children with asthma: 1.39 (95% CI: 0.53‐3.69); interaction *P*‐value = .12].

**Conclusions:**

Asthma was not a risk factor for serious illness among children with influenza. Additional studies are needed to better understand the role of influenza vaccination in preventing serious outcomes among children with asthma.

## 1 INTRODUCTION

Each winter, influenza epidemics cause substantial disease burden for persons of all ages, with an estimated 9‐35 million illnesses resulting in up to 708 000 influenza‐associated hospitalizations in the United States.^1^ School‐aged children typically have the highest attack rates, and children with asthma may be at greater risk for developing severe illness and outcomes associated with influenza infection.^2^, ^3^, ^4^, ^5^ Among children hospitalized with influenza, asthma was the most common underlying condition reported, particularly during the 2009 H1N1 pandemic.^6^, ^7^, ^8^ Some studies have identified asthma as a risk factor for more severe influenza illness, including asthma exacerbations and need for intensive care. This was observed among children hospitalized during the 2009 pandemic, but not consistently among children hospitalized with seasonal influenza infection.^9^, ^10^, ^11^, ^12^

Annual influenza vaccination was first recommended by the US Advisory Committee on Immunization Practices for all children aged 6‐23 months in 2004; by 2008, annual influenza vaccination was recommended for all children aged 6 months‐18 years.^13^ However, recommendations for vaccination of persons in certain groups at higher risk for influenza complications, including asthma, have been in place since the 1960s.^14^ Despite the long‐standing recommendations, data on the effect of influenza vaccination in children with asthma are limited. Most studies use non‐specific outcomes or do not include laboratory‐confirmed influenza infection.^15^, ^16^, ^17^ One study examined vaccine effectiveness during the 2009 pandemic and found no differences in vaccine effectiveness among those with and without asthma.^18^ Similar findings were reported for children and adults with and without asthma during seasonal epidemics, although estimates were lower among those with asthma.^19^

Asthma may have contributed to influenza severity in children during the 2009 pandemic, but it is unclear whether there is a similar increased risk during seasonal epidemics. It is not known whether influenza vaccination reduces the risk of serious influenza illness among children with asthma. In this analysis, we examined whether school‐aged children with asthma were at an increased risk of serious outcomes associated with medically attended, laboratory‐confirmed influenza illness across 10 seasonal epidemics and whether the effect of influenza vaccination on serious outcomes differed between children with and without asthma.

## 2 METHODS

### 2.1 Study population and procedures

This is a secondary analysis of data from annual population‐based studies of influenza vaccine effectiveness conducted by Marshfield Clinic Research Institute in Marshfield, Wisconsin as part of the US Influenza Vaccine Effectiveness (US Flu VE) Network between 2007 and 2018.^20^, ^21^, ^22^, ^23^, ^24^, ^25^, ^26^, ^27^, ^28^ In this community, residents receive nearly all outpatient and inpatient care from the Marshfield Clinic Health System, where both inpatient and outpatient records are accessible.^29^ Each season, patients were actively screened and recruited by research staff during or after a visit for acute respiratory illness. Enrollment was restricted to outpatient departments during all seasons except 2007‐08 and 2008‐09. During those two seasons, patients were enrolled from both the outpatient and hospital setting. Patients were eligible if their acute respiratory illness, defined as cough (all seasons) or fever/feverishness (2007‐08 through 2011‐12), was ≤7 days in duration, and they had not received antiviral medication before enrollment. This analysis was restricted to children aged 5‐17 years with influenza illness confirmed by reverse transcription‐polymerase chain reaction (RT‐PCR). Those with a co‐infection or unsubtypeable influenza A infection were excluded (Figure S1).

At enrollment, parents/guardians reported on their child's illness onset and symptoms, household exposure to smoke (except in 2007‐08), and race/ethnicity. A respiratory sample (nasopharyngeal or combined nasal and oropharyngeal swabs, depending on season) was obtained and tested for influenza by RT‐PCR, including subtype. In addition, information on diagnosis codes, outpatient visits, emergency department (ED) visits, hospitalizations, and medications was extracted from the electronic health record to determine high‐risk status,^19^ number of outpatient visits in the past 12 months (categorized as 0, 1‐4, and ≥5), and receipt of a prescription for antivirals in the 7 days after illness onset. These data were also used to determine asthma status and related serious outcomes, described below.

Study procedures were approved by the Institutional Review Board (IRB) at Marshfield Clinic Health System. Informed consent/assent was obtained from all participants at the time of enrollment into the US Flu VE Network study. Additional analyses for this study were subsequently approved by the IRB with a waiver of informed consent.

### 2.2 Measures

#### 2.2.1 Influenza vaccination

Influenza vaccination history for the enrollment season and all prior seasons was obtained from the immunization registry used by vaccine providers serving the study population.^30^ Children aged ≥9 years were considered vaccinated if they received an influenza vaccine during their enrollment season >14 days before their illness onset. Children aged 5‐8 years were considered fully vaccinated if they (a) received two doses at least 28 days apart during their enrollment season and >14 days before their illness onset or (b) one dose during their enrollment season >14 days before illness onset and ≥2 doses in prior seasons.^31^ Children aged 5‐8 years vaccinated with one dose during their enrollment season >14 days before illness onset and with <2 prior doses were considered partially vaccinated. Participants vaccinated within 14 days prior to symptom onset were excluded (Figure S1).

#### 2.2.2 Asthma

Asthma status was based on asthma‐specific diagnosis codes (ICD‐9 code 493.* prior to October 1, 2015, and ICD‐10 code J45.* on or after October 1, 2015) and prescription medications for asthma in the 2 years prior to enrollment. Step‐up medications and medication classes considered consistent with a diagnosis of mild persistent or more severe asthma were assessed (Table S1). Step 1 asthma medications included short‐acting beta‐agonists. Step 2 or higher medications included anticholinergics, inhaled corticosteroids or inhaled corticosteroid‐beta‐agonist combinations, inhaled long‐acting beta‐agonists, leukotriene modifiers, respiratory smooth‐muscle relaxants (methylxanthines), cromolyn, and immunomodulators. Prescriptions for oral and injectable steroids were not included because they are frequently prescribed for non‐asthma diagnoses.

Asthma status was classified into three groups: confirmed, probable, and no asthma. Children with confirmed asthma had an asthma‐specific diagnosis code and a prescription for a step 2 or higher asthma medication. Children classified with probable asthma had either (a) an asthma‐specific diagnosis code or a prescription for a step 2 or higher asthma medication or (b) an asthma‐specific diagnosis code and a prescription for a step 1 asthma medication. Children without an asthma‐specific diagnosis code and no prescription for a step 1 or higher asthma medication were classified as no asthma. Those without an asthma‐specific diagnosis code, but had a prescription for a step 1 asthma medication, were excluded (Figure S1).

#### 2.2.3 Serious outcome

A serious outcome was defined as hospitalization, ED visit, and/or pneumonia diagnosis within 30 days of symptom onset. A dichotomous composite variable was used for the analysis, and a child with at least one outcome of interest was classified as having a serious outcome. Notes from hospitalizations and ED visits were reviewed by a physician (SCO) to determine whether or not the visits were related to the influenza illness episode. Children were classified as having pneumonia if they received a diagnosis code for pneumonia and a new prescription for antibiotics within 7 days of the pneumonia diagnosis. Those with a code for pneumonia, but no new prescription for antibiotics within 7 days, were excluded (Figure S1).

### 2.3 Statistical methods

Only the first confirmed influenza illness per season per child was included. Enrollments during the 2009‐10 pandemic influenza season were excluded because the seasonal vaccine did not include the A(H1N1)pdm09 strain until the following season. Chi‐square tests were used to compare characteristics of children with and without asthma and children with and without a serious outcome. A multivariable logistic regression model was constructed to estimate the odds of serious outcomes among children with and without asthma. Vaccination status and influenza type/subtype were included in the model a priori; additional confounders were assessed for inclusion using forward selection and an *α* level of .05. Confounders assessed included the following: age group (5‐8 and 9‐17 years), sex, race/ethnicity (non‐Hispanic white, Hispanic, other, unknown), Medicaid coverage in the 12 months prior to enrollment, presence of a high‐risk condition other than asthma in the 2 years prior to enrollment, reported household exposure to smoking, number of outpatient visits in the past 12 months (0, 1‐4, ≥5), illness duration at time of enrollment (0‐2, 3‐4, and 5‐7 days), and receipt of prescription for antivirals within 7 days after onset. To determine whether the effect of vaccination on serious outcome differed between children with and without asthma, a separate multivariable model was established with an interaction term for asthma and vaccination status. For primary analyses, children with possible asthma were excluded and vaccination status was dichotomized by combining fully and partially vaccinated groups. Sensitivity analyses were conducted excluding partially vaccinated children and including children with possible asthma, separately. All statistical analyses were performed using SAS 9.4 (SAS Institute Inc).

## 3 RESULTS

### 3.1 Study population

From 2007‐08 to 2017‐18, there were 1764 medically attended, laboratory‐confirmed influenza infections among school‐aged children enrolled at Marshfield Clinic Health System that met criteria for inclusion in this analysis. Most were aged 9‐17 years (58%), and non‐Hispanic white (90%); 51% were male. There were 790 (45%) influenza B, 765 (43%) influenza A(H3N2), 116 (7%) influenza A(H1N1)pdm09, and 93 (5%) influenza A(H1N1) seasonal infections. The majority of children (1270, 72%) were unvaccinated at the time of influenza illness.

Asthma was confirmed in 287 (16%) children, and 227 (13%) had probable asthma (Figure S1). Children with confirmed asthma differed from children without asthma with regard to several characteristics (Table 1). Children with asthma were more likely to be male (60% vs 49%), have a high‐risk condition other than asthma (13% vs 6%), have ≥5 outpatient visits in the previous year (59% vs 35%), and be vaccinated (42% vs 24%). At the time of enrollment, symptoms reported more often by influenza cases with asthma (vs no asthma) included shortness of breath (49% vs 30%) and wheezing (44% vs 24%). Children with influenza and asthma were more likely to receive antiviral treatment compared with those without asthma (22% vs 7%).

**Table 1:** Characteristics of school‐aged children with influenza by asthma status

	No asthma (N = 1250)		Confirmed asthma (N = 287)		Probable asthma (N = 227)	
	n	%	n	%	n	%
Age (y)						
5‐8	529	42.3	107	37.3	100	44.1
9‐17	721	57.7	180	62.7	127	55.9
Male	610	48.8	171	59.6	122	53.7
Race/ethnicity						
Non‐Hispanic white	1135	90.8	253	88.2	208	91.6
Hispanic	64	5.1	18	6.3	7	3.1
Other	45	3.6	14	4.9	11	4.9
Unknown	6	0.5	2	0.7	1	0.4
Medicaid coverage in past 12 mo	607	48.6	155	54.0	124	54.6
Presence of a high‐risk condition other than asthma	74	5.9	36	12.5	17	7.5
Household exposure to smoke^a^	230	21.2	58	22.9	52	24.9
Number of outpatient visits in past 12 mo						
0	86	6.9	6	2.1	12	5.3
1‐4	732	58.6	113	39.4	120	52.9
≥5	432	34.6	168	58.5	95	41.9
Influenza vaccination status						
Fully vaccinated	281	22.5	117	40.8	68	30.0
Partially vaccinated	21	1.7	3	1.1	4	1.8
Unvaccinated	948	75.8	167	58.2	155	68.3
Influenza season						
2007‐08	160	12.8	33	11.5	18	7.9
2008‐09	295	23.6	52	18.1	35	15.4
2010‐11	45	3.6	9	3.1	12	5.3
2011‐12	54	4.3	12	4.2	6	2.6
2012‐13	187	15.0	49	17.1	47	20.7
2013‐14	52	4.2	7	2.4	8	3.5
2014‐15	139	11.1	35	12.2	27	11.9
2015‐16	21	1.7	6	2.1	5	2.2
2016‐17	140	11.2	36	12.5	30	13.2
2017‐18	157	12.6	48	16.7	39	17.2
Influenza type/subtype						
A(H1N1), seasonal	70	5.6	16	5.6	7	3.1
A(H1N1)pdm09	88	7.0	15	5.2	13	5.7
A(H3N2)	513	41.0	139	48.4	113	49.8
B	579	46.3	117	40.8	94	41.1
Duration of illness at time of enrollment (d)						
0‐2	618	49.4	139	48.4	108	47.6
3‐4	451	36.1	106	36.9	73	32.2
5‐7	181	14.5	42	14.6	46	20.3
Received prescription for antivirals within 7 d after onset	83	6.6	62	21.6	28	12.3
Reported symptoms						
Fatigue	1182	94.6	268	93.4	217	95.6
Fever	1150	92.0	256	89.2	205	90.3
Shortness of breath^b^	326	29.9	125	49.2	77	36.8
Sore throat	1005	80.4	221	77.0	181	79.7
Wheezing	300	24.0	125	43.6	84	37.0

Abbreviations: n, number; %, percentage.

a Missing for n = 217 participants.

b Missing for n = 211 participants.

Children with probable asthma were less likely than children with confirmed asthma to have ≥5 outpatient visits in the previous year (42% vs 59%), be vaccinated (32% vs 42%), receive antivirals (12% vs 22%), and report shortness of breath (37% vs 49%). There were no differences between children with confirmed asthma, probable asthma, or no asthma with regard to race/ethnicity, Medicaid coverage, household exposure to smoking, influenza type/subtype, or duration of illness at enrollment.

### 3.2 Asthma status and serious influenza‐associated outcomes

A serious influenza‐associated outcome within 30 days of illness onset occurred in 104 (6%) children, including 10 hospitalizations, 57 ED visits, and 39 pneumonia diagnoses (two of which were associated with hospitalizations). Hospital admissions occurred 0‐17 days after illness onset (median: 4, interquartile range (IQR): 2‐6), and ED visits ranged from 0 to 29 days after onset (median: 2, IQR: 1‐4).

Children with a serious outcome were more likely to be 5‐8 years old (59% vs 41%, *P* = .0003) and have ≥5 outpatient visits in the previous year (55% vs 38%) (Table 2). The incidence of serious outcomes varied by season, ranging from 2 out of 201 (1%) infections in 2014‐15 to 35 out of 382 (9%) in 2008‐09, but not by influenza type/subtype.

**Table 2:** Characteristics of school‐aged children with influenza by outcome status and odds of serious outcomes

	No serious outcome (N = 1660)		Serious outcome (N = 104)		aOR (95% CI)^a^
	n	%	n	%	
Asthma status					
No asthma	1184	71.3	66	63.5	1.00
Confirmed asthma	269	16.2	18	17.3	1.35 (0.77, 2.35)
Probable asthma	207	12.5	20	19.2	NA
Age (y)					
5‐8	675	40.7	61	58.7	2.34 (1.47, 3.72)
9‐17	985	59.3	43	41.3	1.00
Sex					
Male	858	51.7	45	43.3	1.00
Female	802	48.3	59	56.7	1.70 (1.08, 2.70)
Race/ethnicity					
Non‐Hispanic white	1501	90.4	95	91.3	
Hispanic	86	5.2	3	2.9	
Other	66	4.0	4	3.8	
Unknown	7	0.4	2	1.9	
Medicaid coverage in past 12 mo					
No	833	50.2	45	43.3	
Yes	827	49.8	59	56.7	
High‐risk condition other than asthma					
No	1545	93.1	92	88.5	1.00
Yes	115	6.9	12	11.5	2.75 (1.41, 5.35)
Household exposure to smoke^b^					
No	1144	78.2	63	74.1	
Yes	318	21.8	22	25.9	
Number of outpatient visits in past 12 mo					
0	102	6.1	2	1.9	
1‐4	920	55.4	45	43.3	
≥5	638	38.4	57	54.8	
Influenza vaccination status					
Unvaccinated	1194	71.9	76	73.1	1.00
Vaccinated	466	28.1	28	26.9	0.72 (0.43, 1.23)
Influenza season					
2007‐08	192	11.6	19	18.3	
2008‐09	347	20.9	35	33.7	
2010‐11	61	3.7	5	4.8	
2011‐12	67	4.0	5	4.8	
2012‐13	269	16.2	14	13.5	
2013‐14	64	3.9	3	2.9	
2014‐15	199	12.0	2	1.9	
2015‐16	31	1.9	1	1.0	
2016‐17	197	11.9	9	8.7	
2017‐18	233	14.0	11	10.6	
Influenza type/subtype					
A(H1N1), seasonal	82	4.9	11	10.6	1.90 (0.87, 4.15)
A(H1N1)pdm09	107	6.5	9	8.7	1.46 (0.65, 3.27)
A(H3N2)	724	43.6	41	39.4	0.86 (0.52, 1.42)
B	747	45.0	43	41.4	1.00
Duration of illness at time of enrollment (d)					
0‐2	824	49.6	41	39.4	
3‐4	588	35.4	42	40.4	
5‐7	248	14.9	21	20.2	
Received prescription for antivirals within 7 d after onset					
No	1496	90.1	95	91.3	
Yes	164	9.9	9	8.7	
Reported symptoms					
Fatigue	1571	94.6	96	92.3	
Fever	1511	91.0	100	96.2	
Shortness of breath^c^	495	33.7	33	38.9	
Sore throat	1317	79.3	90	86.5	
Wheezing	472	28.4	37	35.6	

Abbreviations: n, number; %, percentage; NA, not applicable.

a The final multivariate model adjusts for vaccination status, influenza type/subtype, age, sex, and presence of a high‐risk condition other than asthma.

b Missing for n = 217 participants.

c Missing for n = 211 participants.

The odds of a serious outcome did not differ between those with confirmed asthma and those without asthma, after adjusting for vaccination status, influenza type/subtype, age, sex, and presence of a high‐risk condition other than asthma [adjusted odds ratio (aOR): 1.35, 95% confidence interval (CI): 0.77, 2.35] (Table 2). Similar findings were seen in a sensitivity analysis that excluded children who were partially vaccinated (aOR: 1.27, 95% CI: 0.72, 2.25). However, when the sensitivity analysis included children with probable and confirmed asthma, the odds of a serious outcome were significantly higher among children with asthma compared to those without asthma (aOR: 1.55, 95% CI: 1.01, 2.37).

### 3.3 Asthma and effect of vaccination on serious outcomes

The association between vaccination and serious outcomes was not modified by asthma status after adjusting for influenza type/subtype, age, sex, and presence of a high‐risk condition other than asthma. Among children without asthma, the adjusted odds ratio for a serious outcome in vaccinated vs unvaccinated children was 0.55 (95% CI: 0.28‐1.07). Among asthmatic children, this odds ratio was 1.39 (95% CI: 0.53‐3.69) (interaction *P* = .12). Similar results were obtained in two sensitivity analyses. The first one excluded partially vaccinated children, and the second one included children who met the criteria for possible asthma (Table S2).

## 4 DISCUSSION

Across 10 influenza seasons, PCR‐confirmed influenza illness led to few serious outcomes among school‐aged children, similar to the rate of influenza‐related complications (8%) previously reported among a primary care cohort in the UK during the 2009 H1N1 pandemic.^32^ Asthma status was not associated with increased odds of a serious outcome in children with influenza. This is consistent with studies among children hospitalized with laboratory‐confirmed influenza during seasonal epidemics where asthma was not associated with severe illness.^9^, ^33^ Additionally, vaccination did not modify the risk of a serious outcome in children with asthma. This supports findings from the US Flu VE Network showing vaccine effectiveness estimates did not differ among children with and without asthma.^19^

An increased risk for influenza‐related complications among children with asthma was found in a primary care cohort with influenza or influenza‐like illness during the 2009 pandemic, after adjusting for confounders such as age and vaccination status.^32^ Increased asthma exacerbation and serious illness were noted among children hospitalized with pandemic A(H1N1)pdm09 in multiple studies.^9^, ^10^, ^34^, ^35^ In the current study of seasonal influenza, the association between asthma and serious outcomes became significant in a sensitivity analysis that included children with probable asthma, suggesting a potential true association that may be detected in larger samples. It is also possible that serious outcomes among children with asthma may be influenza type/subtype specific. In addition to data from the pandemic, another study found more frequent asthma exacerbations among children with influenza A vs influenza B infection.^34^ We did not observe significant differences in the odds of serious outcomes by influenza type/subtype, though were limited by sample size. Larger studies are needed to better understand the relationship between asthma and serious outcomes during seasonal epidemics by influenza subtype.

There was no difference in the odds of a serious outcome between vaccinated and unvaccinated children with asthma in this study. A clinical trial among similar aged children with asthma in the Netherlands also did not see a reduction in asthma exacerbations caused by influenza among vaccinated children compared to unvaccinated children.^36^ However, most children in this study likely had mild‐to‐moderate asthma. Influenza vaccine may provide protection against influenza infection,^19^, ^37^, ^38^ but does not appear to prevent serious outcomes among influenza infected children with asthma. However, these conclusions may not apply to children with more severe asthma.

Strengths of this study include systematic recruitment and sample collection from a community cohort with medically attended, laboratory‐confirmed influenza, access to a validated immunization registry, and complete capture of ED visits and hospital admissions from the electronic health record. This study also has important limitations. During most seasons, enrollments occurred in the primary care and urgent care setting, and most children who initially presented to the ED with severe influenza illness were not enrolled. As a result, the study enrollments may have been biased toward children with stable, well‐controlled asthma. We were unable to quantify baseline asthma severity, a potential source of confounding with vaccination status and serious outcome. There were few serious outcomes associated with influenza infection in this population, and we used a composite outcome due to the limited number of events in each category. The most common serious outcome was ED visit, but this is not a direct measure of illness severity. Emergency department utilization was likely influenced by parental choice and other factors not related to severity of illness. Pneumonia diagnoses were not validated by radiographic findings, and non‐differential misclassification may have occurred. Finally, this study was conducted in a rural Midwestern health system with little racial or ethnic diversity. The findings may not be generalizable to more diverse urban populations.

## 5 CONCLUSIONS

Asthma is the most common underlying condition among children hospitalized with influenza^5^, ^6^ and is often cited as a risk factor for hospitalization among both children and adults with influenza. In this multi‐season study, asthma was not associated with a serious outcome among children with influenza, and influenza vaccination did not modify the risk of a serious outcome. However, asthma is common among children hospitalized for influenza, and influenza‐attributable hospitalizations occur more often among children aged 6‐23 months with asthma compared to those without.^5^ Additional studies are needed to better understand the role of influenza vaccination in preventing these serious outcomes among children with asthma. In particular, children with severe or poorly controlled asthma merit further investigation to understand the effects of influenza illness and vaccination.

## CONFLICT OF INTEREST

HQM and KEH report research support from Seqirus for unrelated studies. All other authors report no conflicts of interest.

## Data Availability

The data that support the study conclusions are unavailable for public access because informed consent to share said data (beyond the research team) was not obtained from study participants.
