# Supplementary material for: Serious outcomes of medically attended, laboratory‐confirmed influenza illness among school‐aged children with and without asthma, 2007‐2018
Source: Influenza Other Respir Viruses. 2020 Jan 14;14(2):173–81. doi: 10.1111/irv.12710 (PMC7040974; doi:10.1111/irv.12710)
Supplement: Supplementary file 1 [file IRV-14-173-s001.docx]

**Supplemental Figure S1. Study Flow Diagram**

**Supplemental Table S1. Medications Utilized to Classify Asthma Status**

| **Generic Name** | **Brand Name(s)** | **Drug Class** | **Level** |
| --- | --- | --- | --- |
| Albuterol Sulfate | ProAir, AccuNeb | Short-Acting Beta-Agonists | Step 1 |
| Albuterol Sulfate | VoSpire ER | Long-Acting Beta-Agonists | Step 2+ |
| Albuterol Sulfate HFA | ProAir HFA, Proventil HFA, Ventolin HFA | Short-Acting Beta-Agonists | Step 1 |
| Beclometasone Dipropionate HFA | QVAR | Inhaled Corticosteroids | Step 2+ |
| Benralizumab | Fasenra | Immunomodulators | Step 2+ |
| Budesonide | Pulmicort | Inhaled Corticosteroids | Step 2+ |
| Budesonide + Formoterol | Symbicort | Combination Medications | Step 2+ |
| Ciclesonide | Alvesco | Inhaled Corticosteroids | Step 2+ |
| Cromolyn Sodium | Intal | Cromolyn | Step 2+ |
| Flunisolide | Aerobid | Inhaled Corticosteroids | Step 2+ |
| Fluticasone + Salmeterol | Advair | Combination Medications | Step 2+ |
| Fluticasone Furoate | Arnuity | Inhaled Corticosteroids | Step 2+ |
| Fluticasone Furoate + Vilanterol | Breo | Combination Medications | Step 2+ |
| Fluticasone Propionate | Flovent | Inhaled Corticosteroids | Step 2+ |
| Fluticasone Propionate + Salmeterol | AirDuo | Combination Medications | Step 2+ |
| Formoterol Fumarate | Foradil | Long-Acting Beta-Agonists | Step 2+ |
| Levalbuterol HCl | Xopenex, Xopenex HFA | Short-Acting Beta-Agonists | Step 1 |
| Mepolizumab | Nucala | Immunomodulators | Step 2+ |
| Mometasone | Asmanex | Inhaled Corticosteroids | Step 2+ |
| Mometasone Furoate HFA | Asmanex HFA | Inhaled Corticosteroids | Step 2+ |
| Mometasone + Formoterol | Dulera | Combination Medications | Step 2+ |
| Montelukast | Singulair | Leukotriene Modifiers | Step 2+ |
| Omalizumab | Xolair | Immunomodulators | Step 2+ |
| Reslizumab | Cinqair | Immunomodulators | Step 2+ |
| Salmeterol Xinafoate | Serevent | Long-Acting Beta-Agonists | Step 2+ |
| Theophylline | Uniphyl | Methylxanthines | Step 2+ |
| Tiotropium | Spiriva | Anticholinergics | Step 2+ |
| Triamcinolone Acetonide | Azmacort | Inhaled Corticosteroids | Step 2+ |
| Zafirlukast | Accolate | Leukotriene Modifiers | Step 2+ |
| Zileuton | Zyflo | Leukotriene Modifiers | Step 2+ |

**Supplemental Table S2. Sensitivity Analyses: Effect of Vaccination on Serious Influenza-Associated Outcomes among Children with and without Asthma**

|  | **No Asthma** | | **Asthma** | | **Interaction p-value** |
| --- | --- | --- | --- | --- | --- |
| **Excluding Partially Vaccinated** | **n/N** | **aOR (95% CI)^1^** | **n/N** | **aOR (95% CI)^1^** | 0.1804 |
| Unvaccinated | 55/948 | 1.00 | 9/167 | 1.00 |  |
| Vaccinated | 10/281 | 0.55 (0.27, 1.11) | 8/117 | 1.27 (0.47, 3.44) |  |
| **Including Possible Asthma** | **n/N** | **aOR (95% CI)^1^** | **n/N** | **aOR (95% CI)^1^** | 0.0770 |
| Unvaccinated | 55/948 | 1.00 | 21/322 | 1.00 |  |
| Vaccinated^2^ | 11/302 | 0.56 (0.28, 1.08) | 17/192 | 1.31 (0.67, 2.58) |  |

Abbreviations: n=number of serious outcomes; N=sample size; aOR=adjusted odds ratios; 95% CI=95% confidence interval.

^1^Calculated from a model that includes an asthma status*vaccination status interaction term and adjusts for influenza type, categorical age, sex, and presence of a high-risk condition other than asthma.

^2^Includes partially vaccinated and fully vaccinated children.
